# C21 steroid-enriched fraction refined from *Marsdenia tenacissima* inhibits hepatocellular carcinoma through the coordination of Hippo-Yap and PTEN-PI3K/AKT signaling pathways

**DOI:** 10.18632/oncotarget.22833

**Published:** 2017-11-30

**Authors:** Yu Zhang, Kaiqiang Li, Youmin Ying, Bingyu Chen, Ke Hao, Boxu Chen, Yu Zheng, Jianxin Lyu, Xiangming Tong, Xiaopan Chen, Ying Wang, Zhajun Zhan, Wei Zhang, Zhen Wang

**Affiliations:** ^1^ Research Center of Blood Transfusion Medicine, Education Ministry Key Laboratory of Laboratory Medicine, Zhejiang Provincial People's Hospital, People's Hospital of Hangzhou Medical College, Hangzhou 310014, China; ^2^ College of Pharmaceutical Science, Zhejiang University of Technology, Hangzhou 310014, China; ^3^ Key Laboratory of Tumor Molecular Diagnosis and Individualized Medicine of Zhejiang Province, Zhejiang Provincial People's Hospital, People's Hospital of Hangzhou Medical College, Hangzhou 310014, China; ^4^ Department of Reproductive Endocrinology, Zhejiang Provincial People's Hospital, People's Hospital of Hangzhou Medical College, Hangzhou 310014, China

**Keywords:** hepatocellular carcinoma, Marsdenia tenacissima, Hippo-YAP signaling pathway, PI3K/AKT signaling pathway, C21 steroids

## Abstract

*Marsdenia tenacissimae* extraction (MTE), a traditional herbal medicine, has exhibited anti-tumor effects on a variety of cancers. However, its effectiveness and the mechanism of action in Hepatocellular carcinoma (HCC) has not been fully understood. In the present study, we demonstrate that C21 steroid-enriched fraction from MTE, which contains five main C21 steroids (FR5) exhibits obvious pharmacological activities on HCC cells *in vitro and in vivo*. FR5 induces apoptosis and inhibits proliferation and migration of HepG2 and Bel7402 cells in a dose and time dependent manner. Furthermore, in HCC cells, we found that FR5 inhibits Hippo pathway, leading to inactivation of YAP and increase of PTEN. Enhanced PTEN results in the inhibition of PI3K/AKT signaling pathway, inhibiting cell proliferation by FR5 and FR5-induced apoptosis. Moreover, it was proved that FR5 treatment could inhibit tumor growth in a HCC xenograft mouse model, and immunohistochemistry results showed FR5 treatment resulted in down-regulation of Bcl-2 and YAP, and up-regulation of PTEN and PI3K. Taken together, we found that FR5 effectively inhibits proliferation and induces apoptosis of HCC cells through coordinated inhibition of YAP in the Hippo pathway and AKT in the PI3K-PTEN-mTOR pathway, and suggest FR5 as a potential therapy for HCC.

## INTRODUCTION

Hepatocellular carcinoma (HCC) is the fifth most prevalent cancer and the third leading cause of cancer-related mortality worldwide [1]. Due to high incidence of tumor recurrence, frequent intrahepatic spread and extrahepatic metastasis during the initial diagnosis, the 5-year recurrence rate is approximately 70% [2]. HCC patients are often at an advanced stage of disease at the time of diagnosis and can only be treated with chemotherapy due to the poor response to hepatic resection and liver transplant therapy as treatment, so its chemotherapeutic drugs have become a hotspot of research [3]. At present, the main chemotherapeutic drugs of HCC are cisplatin, 5-fluorouracil, doxorubicin and mitomycin. Therefore, the development of more effective drugs and therapeutic approaches to HCC is still in urgent need.

*Marsdenia tenacissima* is an asclepiadaceous plant widely produced in south of China [4]. *Marsdenia tenacissima* extraction (MTE, also called Xiao-Ai-Ping injection) is a traditional herbal medicine with significant anti-tumor effect, as adjuvant therapy after chemotherapy and radiation therapy [5], particularly for esophageal cancer, lung cancer, leukemia and hepatocellular carcinoma [6–9]. More than forty C21 steroidal glycosides have been isolated from MTE [10], and studies demonstrate that components of C21 steroids are specific cytotoxic to various cancer cells [11] through various mechanisms, such as inhibition of tumor growth, induction of apoptosis and cell cycle arrest, and reverse of multidrug resistance [12–13]. MTE is effective in combination with other chemotherapeutic drugs. In order to improve clinical efficacy and application, the underlying molecular mechanism and the active components of MTE require further study. Hence, FR5 was purified from MTE by HPLC to make more efficient and less toxic effect.

Although the anti-tumor effect has been identified, the underlying molecular mechanism of MTE in HCC has not been fully understood. Han et al. [14] had showed that MTE inhibited the growth of gefitinib resistant non-small lung cancer cell through blocking PI3K/AKT/mTOR pathway. Our previous study had proved MTE inhibits human acute T cell leukemia cells through enhancing PTEN pathway [9]. The liver-specific knockout of PTEN [15] and the activation of the YAP pathway by knockout of upstream negative regulators both can induce HCC [16], while the nature of the interactions among these pathways has remained poorly understood. Further, it has been reported that YAP mediates crosstalk between the Hippo and PI3K/AKT pathways by regulating PTEN in MCF10A and Hela cells [17]. It is amazed whether YAP affecting PI3K/AKT pathway via PTEN pathway in FR5-treated HCC.

In the present study, a C21 steroid-enriched fraction, FR5, was prepared from MTE by column chromatography. This study aims to evaluate the anti-HCC activities of FR5 and define its associated molecular mechanisms to further understand its anti-tumor effect. We found that FR5 potently inhibited the proliferation and migration and promoted apoptosis in HCC cells. Further mechanical studies identify a coordination between Hippo-YAP and PTEN-PI3K/AKT signaling pathways for the effects of FR5 on HCC therapy.

## RESULTS

### Preparation and chemical analysis of FR5

**Figure 1:**
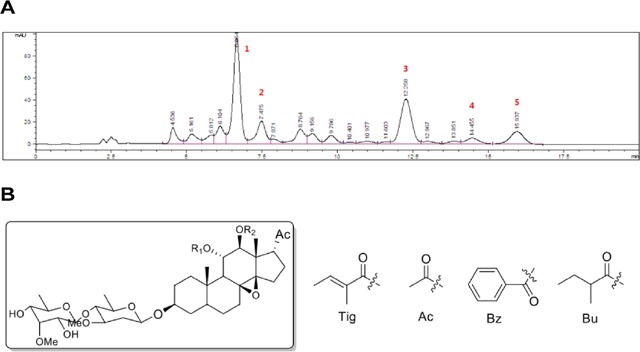
Preparation and chemical analysis of FR5 **(A)** HPLC profiles of FR5 (Column: YMC Triart C18 column (250 × 4.6 mm); Mobile phase: CH_3_OH/H_2_O = 80/20; Flow rate: 1 mL/min; Wavelength: 210 nm; Temperature: 28 °C). **(B)** Five representative C21 steroids identified in FR5.

FR5 was separated from the MTE by column chromatography on silica gel eluted with gradient petroleum ether and EtOAc. Furthermore, five compounds 1∼5 were isolated from FR5 by preparative high performance liquid chromatography (HPLC) (Figure 1A) and identified by nuclear magnetic resonance (NMR) analyses and comparison with the literatures to be: tenacissoside G [18], tenacissoside I [18], marsdenoside A [19], marsdenoside B [19], and marsdenoside C [19] (Figure 1B, Table 1). All the isolated compounds belong to C21 steroids, the characteristic constituents of *Marsdenia tenacissima*.

**Table 1:** The basic information of the five principal components

Peak no.	Retention time (min)	Chemical structure	Chemical name
1	6.654	R_1_ = Tig, R_2_ = Ac	Tenacissoside G [18]
2	7.475	R_1_ = Bz, R_2_ = Ac	Tenacissoside I [18]
3	12.258	R_1_ = Bu, R_2_ = Tig	Marsdenoside A [19]
4	14.455	R_1_ = Tig, R_2_ = Tig	Marsdenoside B [19]
5	15.937	R_1_ = Bu, R_2_ = Bz	Marsdenoside C [19]

Five compounds 1∼5 were isolated from FR5 by preparative high performance liquid chromatography (HPLC) and identified by nuclear magnetic resonance (NMR) analyses and compared with the literatures.

### FR5 suppresses the proliferation of Bel7402 and HepG2 cells

**Figure 2:**
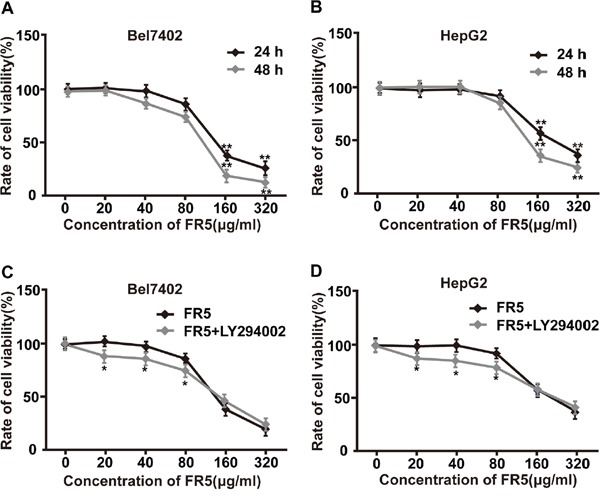
Effect of FR5 on the proliferation of Bel7402 and HepG2 cells **(A, B)** Bel7402 and HepG2 cells were seeded into 96-well plates at a density of 5 × 10^3^ cells/well and then treated with FR5 at concentrations of 0, 20, 40, 80, 160 and 320 μg/ml for 24 and 48 h, and the cell viability rates were determined using MTS assay. **(C, D)** Bel7402 and HepG2 cells were treated with FR5 at concentrations of 0, 20, 40, 80, 160 and 320 μg/ml for 24 h with or without LY294002 pretreatment (25 μmol/L), and cell viability rates were examined and compared. The data are expressed in terms of percent of control cells as the means ± SD. The experiments were repeated at least three times. ^*^p<0.05; ^**^p<0.01 vs. control group.

We examined the *in vitro* cell growth inhibition of FR5 on HCC cell lines and non-cancerous cell lines by using MTS assay. The proliferation of Bel7402 and HepG2 cells was notably decreased after FR5 treatment for 24 h or 48 h and the growth inhibitory rate of FR5 in hepatoma clone strains was in a dose-dependent manner, ranging 20−320 μg/ml FR5 treatment, compared with non-FR5-treated cells (Figure 2A, 2B). In detail, FR5 showed higher cytotoxicity toward the Bel7402 cells. The IC50 concentration of that at 24 h and 48 h was 118.8 and 100.6 μg/mL, respectively (Figure 2A). Consistently, the IC50 value of FR5 was 138.2 and 107.4 μg/mL for 24 and 48 h in HepG2 cells, respectively (Figure 2B). Specifically, the FR5-induced inhibitory effect markedly increased between concentrations of 80 μg/ml and 160 μg/ml (p < 0.01). Therefore, 160 μg/ml and 80 μg/ml of FR5 were used in subsequent experiments. Meanwhile, the IC50 value of Xiao-ai-ping injection was 283.3 μg/ml for 24h in Bel7402 cells (Supplementary Figure 1), which was larger than that of FR5. However, FR5 showed a lower cytotoxicity toward the L02 cells and HEK293 cells, its IC50 for 24 h were 306.9 μg/ml and 422.1 μg/ml, respectively, much higher than that in Bel7402 and HepG2 cells (Supplementary Figure 2). Similarly, the RTCA profiling showed that FR5 can inhibit the proliferation of Bel7402 and HepG2 cells in a dose-dependent manner (Supplementary Figure 3). In order to further understand the effect of FR5, Bel7402 and HepG2 cells were exposed to different concentrations of FR5 for 24 h with or without the pretreatment of 2-(4-morpholinyl)-8-phenyl-chromone (LY294002, a PI3K inhibitor). Our results showed that LY294002 increased FR5-induced inhibition at the lower concentration than that of FR5 treated only at ranging 20−80 μg/ml FR5 treatment (Figure 2C, 2D, p < 0.05), which suggest that FR5 reduce the viability of Bel7402 and HepG2 cells, and LY294002 has synergistic effect on FR5 anti-proliferation.

### FR5 enhances the apoptosis of Bel7402 and HepG2 cells

**Figure 3:**
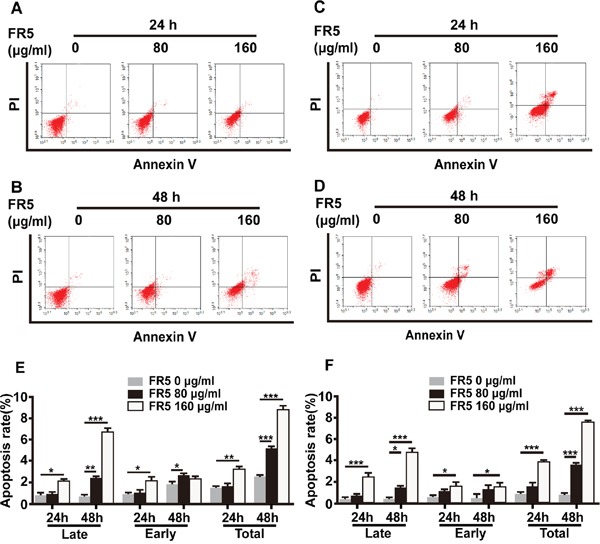
Apoptosis-induced effect of FR5 in Bel7402 and HepG2 cells Bel7402 and HepG2 cells were planted into 6-well plates at 2 × 10^5^ cells/well, treated with FR5 at 0 (Ctrl), 80 and 160 μg/ml for 24 and 48 h, stained with Annexin V-FITC/PI, and detected by flow cytometry. **(A, B)** The apoptosis rate of HepG2 cells at 24 h and 48h of FR5 incubation, respectively. **(C, D)** The apoptosis rate of Bel7402 cells at 24h and 48 h of FR5 incubation, respectively. **(E, F)** Histogram shows the difference of HepG2 and Bel7402 apoptotic cells (%) between time and doses. Results were presented as mean ± SD, and the error bars represent the SD of three independent experiments. ^*^p<0.05; ^**^p<0.01; ^***^p<0.001 *vs* control group.

In order to study inhibitory activity of FR5, a flow cytometric analysis method was used to evaluate FR5-induced apoptosis after dual staining of cells with AnnexinV-FITC and propidium iodide. Apoptotic cell death is determined in terms of early- or late-stage apoptotic cells, which are shown in the lower right and upper right quadrants of the FACS histograms, respectively. FR5 induced high levels of apoptosis in Bel7402 cells. The percentage of total apoptotic cells in Bel7402 cells after treatment with FR5 (80 and 160 μg/ml) was as follows: 3.25 ± 1.3%, 6.78 ± 0.41% for 24h (Figure 3C) and 7.06 ± 0.63%, 14.28 ± 0.78 % for 48h (Figure 3D) compared with the respective controls. Similar tendency of apoptosis was observed in HepG2 cells. As shown in Figure 3A and 3B, the percentage of total apoptotic cells in HepG2 cells after treatment with FR5 (80 and 160 μg/ml) was as follows: 1.36 ± 0.1%, 3.13 ± 0.27% for 24h (Figure 3A) and 5.34 ± 0.57%, 8.89 ± 0.45 % for 48h (Figure 3B). Further quantification analysis of apoptosis HepG2 and Bel7402 are shown in Figure 3E and 3F, that the late- or total apoptosis of them were obviously induced ranging from 80 to 160 μg/ml FR5. Taken together, FR5 ranging from 80 to 160 μg/ml increased the apoptosis of Bel7402 and HepG2 cells at 24 h or 48 h treatment.

### FR5 inhibits the migration of Bel7402 and HepG2 cells

**Figure 4:**
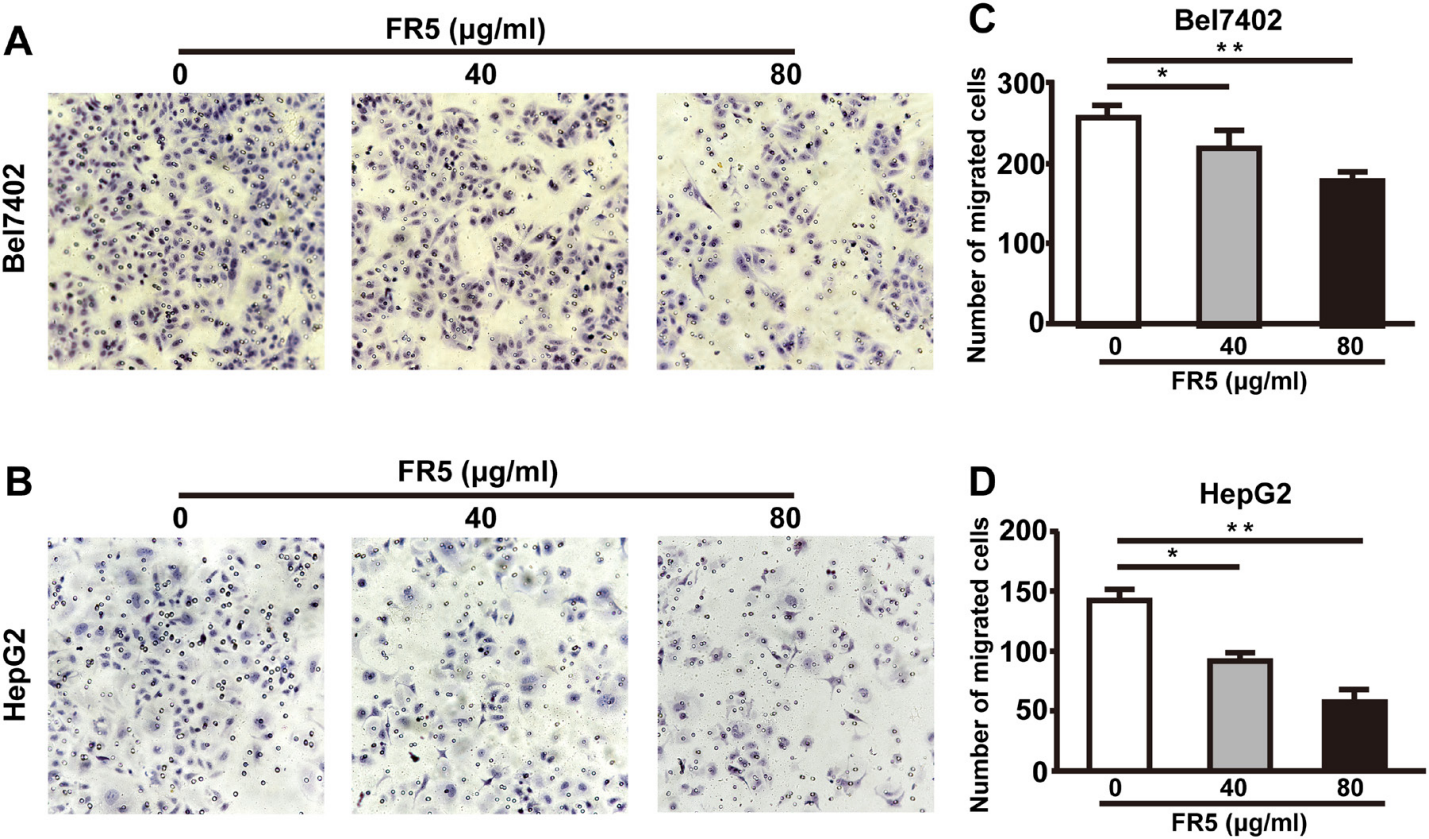
Effects of FR5 on Bel7402 and HepG2 cell migration Cell penetration through to the lower surface were stained with crystal violet and photographed under a light microscope. **(A, B)** Bel7402 and HepG2cells were cultured with FR5 at 0 (Ctrl), 40 and 80 μg/ml for 24 h, and representative pictures of migrated cells are shown. **(C, D)** The cell migration was quantified by counting migrated cells in five randomly selected fields at 24 h after seeding and presented as mean ± SD; ^*^p<0.05; ^**^p<0.01 vs. control group.

To further investigate the effects of FR5 on Bel7402 and HepG2 cell migration, we performed Transwell cell migration assay. After incubation with FR5 (40 and 80 μg/ml) for 24 h, the cell vertical migration distance through the Transwell chamber was significantly decreased compared with the blank control cells, suggesting that FR5 inhibits the migratory capabilities of Bel7402 and HepG2 cells in a dose-dependent manner (Figure 4A, 4B, p < 0.01). Furthermore, FR5 at concentrations of 40 and 80 μg/ml did not significantly reduce the viability of Bel7402 and HepG2 cells as shown in Figure 2, which excluded the influence of FR5-inducd reduction of cell viability to the experimental results of cell migration. These consistent results suggested that FR5 can effectively reduce the metastatic potentials of the Bel7402 and HepG2 cells.

### FR5 dose-dependently inhibits Hippo-YAP and PTEN-PI3K/AKT signaling pathways in hepatocellular carcinoma cells

**Figure 5:**
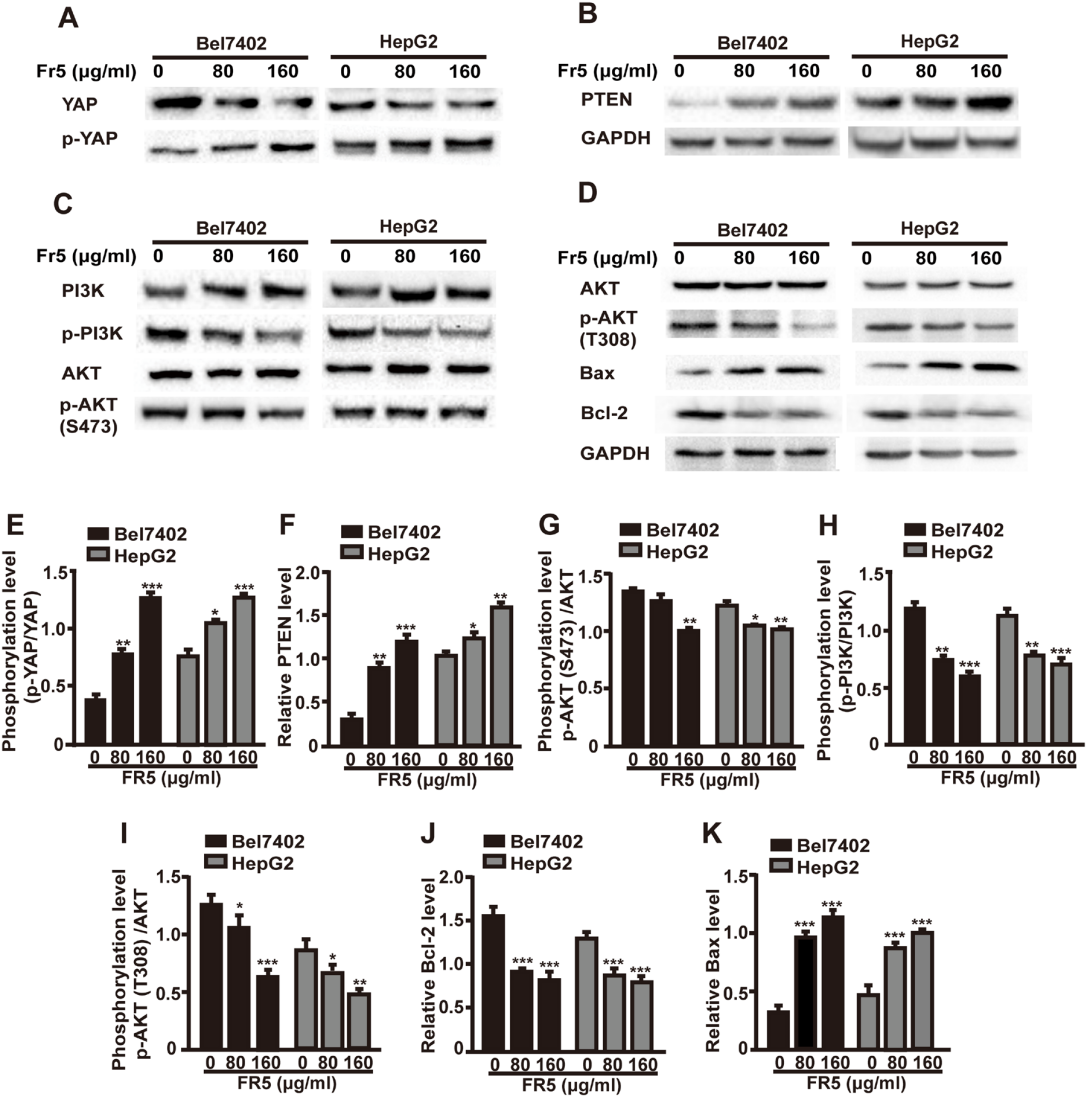
FR5 suppresses Hippo-YAP and PTEN/PI3K/AKT pathways in Bel7402 and HepG2 cells Bel7402 and HepG2 cells were incubated with FR5 at concentrations of 0 (Ctrl), 80 and 160μg/ml for 24 h, and expressions factors were examined by Western blot. **(A)** Membranes were probed with YAP, phospho-YAP. **(B)** Membranes were probed with PTEN. **(C)** Membranes were probed with PI3K, phospho-PI3K, AKT and phospho-AKT(S473). **(D)** Membranes were probed AKT, phospho-AKT(T308), Bcl-2 and Bax. **(E-K)** Quantitative analysis of the relative PTEN (F), Bcl-2 (J), BAX (K), p-YAP/YAP (E), p-AKT(S473)/AKT (G), p-PI3K/PI3K (H) and p-AKT(T308)/AKT (I) as shown in (A-D). Representative blots are presented and corresponding densitometric analyses are shown as mean ± SD from three independent experiments. GAPDH was used as internal control. ^*^p<0.05; ^**^p<0.01; ^***^p<0.001 compared with the control group.

YAP is the main acting downstream target of the Hippo pathway and mediates organ growth and maintaining the balance of proliferation and apoptosis, and the YAP excessive expression leads the proliferation of tumor cell [20]. It was interesting to shed light on whether FR5 modulates the Hippo pathway, the total and the phosphorylation protein level of YAP was examined. We were delighted to find that FR5 could dose-dependently elevate the phosphorylation levels of YAP with obvious reduction of the total YAP expression levels in Bel7402 and HepG2 cells (Figure 5A, 5E). The PI3K/AKT signaling pathway plays a critical role in cell growth, apoptosis, invasion and metastasis, and AKT is the central mediator of the PI3K/AKT pathway [21, 22]. Further study detected whether FR5 promoted the apoptosis of Bel7402 and HepG2 cells through PI3K/AKT pathways. Results showed the phosphorylation level of AKT and PI3K were significantly downregulated in a dose-dependent manner, with no obvious changes of the total AKT and PI3K level in the FR5-treated Bel7402 and HepG2 cells, compared with the control group (Figure 5C, 5D, 5G-5I). We also checked the expression of apoptosis regulators and found that the expression level of Bcl-2 was down-regulated, but the level of Bax was up-regulated in the FR5-treated Bel7402 and HepG2 cells (Figure 5D, 5J, 5K), compared with control group. Several studies have identified the tumor suppressor PTEN, an upstream negative regulator of AKT, as a critical mediator of YAP in AKT regulation [17, 23]. As shown in Figure 5B, the PTEN protein level was significantly increased by FR5 treatment. The expression levels of PTEN in 160 μg/ml FR5 treated Bel7402 and HepG2 cells were 4.22-fold and 1.48-fold higher than in control cells, respectively (Figure 5B, 5F, p<0.01). These data indicated that FR5 promoted the death of hepatoma carcinoma cell by inhibiting Hippo-YAP and PTEN-PI3K/AKT signaling pathways.

### FR5 is more potent than LY294002 in inhibition of the proliferation of hepatocellular carcinoma cells by YAP/PTEN/PI3K/AKT signaling pathway

**Figure 6:**
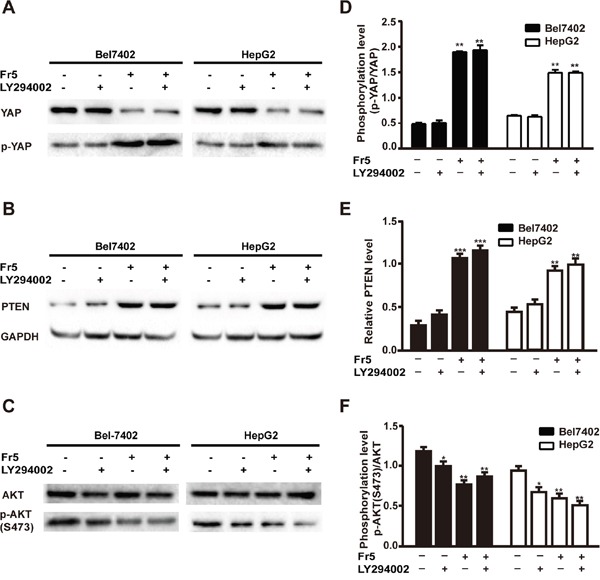
Effects of FR5, LY294002 or combination on Hippo-YAP and PTEN/PI3K/AKT pathways in Bel7402 and HepG2 cells Bel7402 and HepG2 cells were treated with FR5 (160 μg/ml) and LY294002 (25μmol/L) used singly or in combination for 24 h, pretreatment of LY294002 was followed by FR5 in combined treatment. **(A)** Membranes were probed with YAP, phospho-YAP. **(B)** Membranes were probed with PTEN. **(C)** Membranes were probed with AKT and phospho-AKT(S473). **(D-F)** Quantitative analysis of the relative PTEN (E), p-YAP/YAP (D) and p-AKT(S473)/AKT (F) as shown in (A-C). Each bar represented mean±SD of three separate experiments. GAPDH was used as internal control. ^**^p < 0.01 compared with the control group.

In order to further elucidate the apoptosis mechanism induced by FR5, Hippo-YAP and PTEN/PI3K/AKT pathways were examined in Bel7402 and HepG2 cells exposed to FR5 or LY294002 alone or in combination by Western blot analysis. Previous studies revealed that LY294002 inhibits cell growth and induces apoptosis in various types of cancer by decreasing the phosphorylation of AKT [24, 25]. In the present study, FR5 inhibited PI3K/AKT signaling pathway through downregulation of YAP and upregulation of p-YAP (Figure 6A, 6D) and PTEN (Figure 6B, 6E). Both LY294002 and FR5 could inhibit PI3K/AKT pathway, but the degree of change was greater for FR5. Co-treatment of FR5 with LY294002 resulted in no significant difference in decreasing p-AKT(S473)/AKT as compared to the group treated with FR5 alone (Figure 6C, 6F), indicating no stacking effect (Figure 6A, 6B, 6C). However, treatment with FR5 alone or in combination with LY294002 induced a more significant reduction in the phosphorylation of AKT in Bel7402 and HepG2 cells as compared to the group treated with LY294002 alone (Figure 6C), which was consistent with the results of the MTS assays. Taken together, these results suggest that changes in PI3K/AKT axis may contribute partly to the inhibitory effects of FR5 on proliferation and migration in Bel7402 and HepG2 cells.

### The inhibition of FR5 on PTEN/PI3K/AKT pathway was terminated by YAP knockdown on hepatocellular carcinoma cells

**Figure 7:**
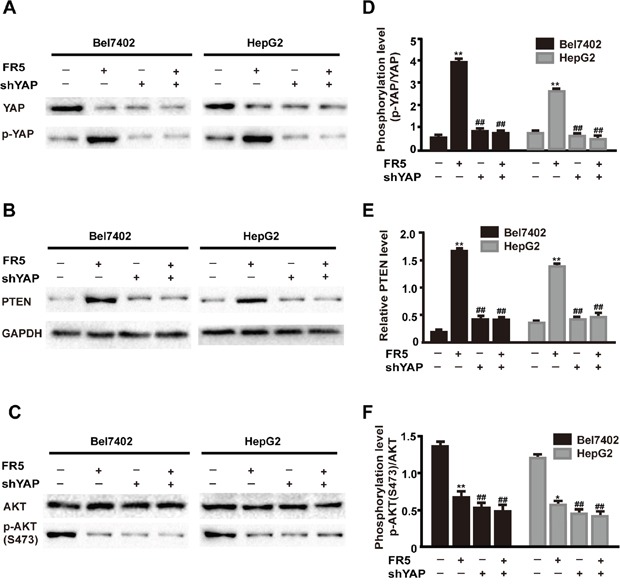
FR5 induces coordination between Hippo and PI3K/AKT inBel7402 and HepG2 cells Bel7402 and HepG2 cells were transfected with shYAP for 24h and then added 160 μg/ml FR5-containing medium for another 24 h. **(A)** Membranes were probed with YAP, phospho-YAP. **(B)** Membranes were probed with PTEN. **(C)** Membranes were probed with AKT and phospho-AKT(S473). **(D-F)** Quantitative analysis of the relative PTEN (E), p-YAP/YAP (D) and p-AKT(S473)/AKT (F) as shown in (A-C). Each bar represented mean ± SD of three separate experiments. GAPDH was used as internal control. ^*^p < 0.05; ^**^p < 0.01; compared with the control group. ^##^p < 0.01compared with FR5 treatment.

Several functional studies have provided evidence for a crosstalk between the Hippo and PI3K-mTOR pathways in Drosophila and identified the tumor suppressor PTEN as a critical mediator of YAP in mTOR regulation [17, 26, 27]. Given the factor that YAP mediates the predominant effector of Hippo, the effect of FR5 on the coordination of these two pathways was examined by silencing YAP expression. Results showed the effect of YAP shRNA1 is better than that of YAP shRNA2 (Supplementary Figure 4). Therefore, YAP1 was used in subsequent experiments. To confirm that transfection with YAP shRNA can significantly decrease YAP expression, the levels of YAP protein were determined in control and FR5-exposed HepG2 and Bel7402 cells transfected with YAP shRNA or control shRNA. As shown in Figure 7A, while transfection with control shRNA did not alter intracellular YAP expression compared with empty control, silencing of YAP using shRNA resulted in a markedly reduced of YAP protein levels with or without FR5 presence. To investigate whether alteration of YAP contribute to the FR5-mediated impairment in phosphorylation of AKT, the expression levels of protein for YAP were examined in the cell line stably transfected with shRNA in the presence or absence of FR5 treatment. In YAP-knockdown cells, Treated with FR5 further decreased YAP expression as compared to the cells without treatment with FR5. As expected, the phosphorylation levels for YAP was also impaired in FR5 treated cells compared with cells without treatment with FR5 (Figure 7A, 7D). As shown in Figure 7C, Western Blots show YAP knockdown could block the decrease of AKT phosphorylation levels, and no obvious changes were found in total AKT in Bel7402 and HepG2 cells treated with FR5 (Figure 7C, 7F). Looking further upstream in the PI3K/AKT pathway, it was observed that FR5-induced upregulation of PTEN was abolished in the YAP-knockdown cells (Figure 7B, 7E).Altogether, these results indicate the effect of FR5 on PI3K/AKT pathway was modulated by Hippo-YAP pathway through the mediation of tumor suppressor PTEN.

### FR5 inhibits the growth of Bel7402 cell xenograft tumors in nude mice

**Figure 8:**
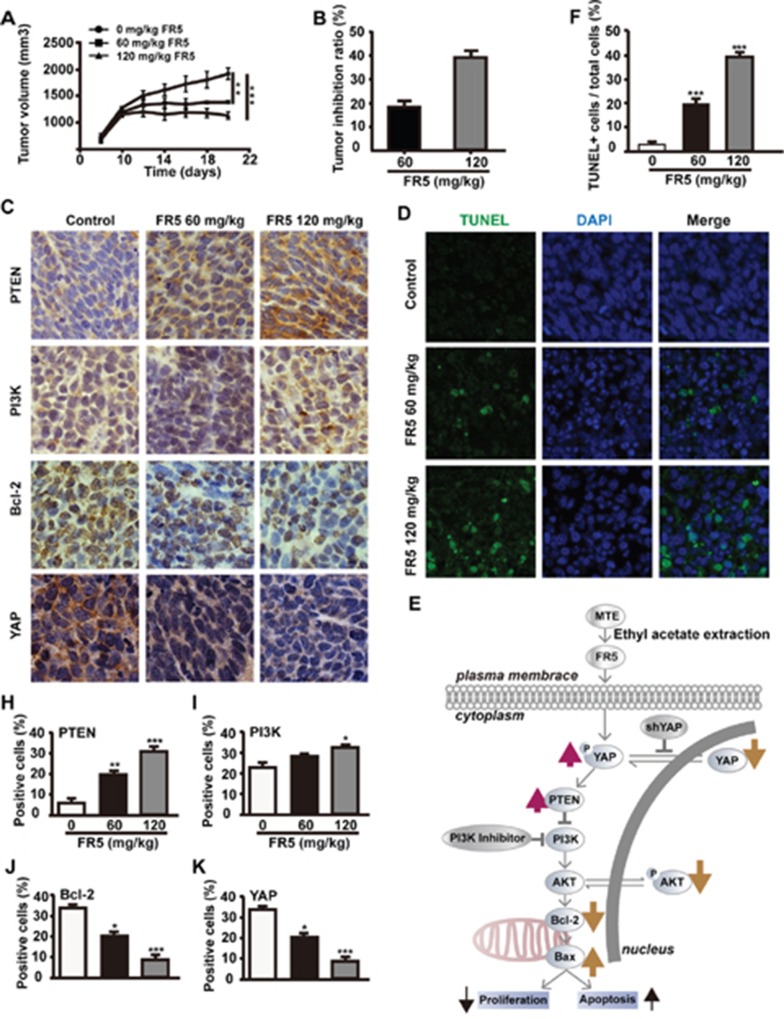
FR5 inhibits the growth of Bel7402 xenograft tumors in nude mice Bel7402 cells were subcutaneously injected into the mouse right axillary fossa to establish xenograft models. The mice were treated with FR5 at the indicated concentrations. **(A, B)** After treatments, all mice were observed and the tumor volume was measured and compared. After treatments, the tumors were harvested and weighed. **(C)** Expression of the factors involved in cancer cell proliferation after FR5 treatments in the Bel7402 xenograft tumors. The expressions of PI3K, Bcl-2, PTEN and YAP in xenograft tumors were analyzed by immunohistochemistry (original magnification 200×). **(H-K)** Quantitative analysis of the percentages of immunohistochemistry positive cells over total cells within 5 medium-power fields under microscope shown in (C). **(D)** Detection of apoptotic cells in tumor tissue was performed by transferase-mediated dUTP nick end labeling (TUNEL) assay. **(F)** Quantitative analysis of the percentages of TUNEL positive cells over total cells in one filed shown in (D). **(E)** Working model of signaling pathways induced by FR5 and the effects of FR5 in HCC cells. ^*^p < 0.05; ^**^p < 0.01; ^***^p < 0.001 versus the control group.

The xenograft tumor model of Bel7402 cell was established in BALB/c nude mice to evaluate the anti-cancer effect of FR5. Significant changes were observed in xenograft tumors of nude mice after the treatment of FR5. Tumor inhibitory rates were 18.3% and 38.8% at the doses of 60 and 120 mg/kg, respectively in the FR5-treated group. Ten days post-treatment, mice were sacrificed, the tumors removed and weighed, and the expression of the corresponding factors were further examined by immunohistochemistry. Compared with the blank control group, the tumor volume of both 60 and 120 mg/kg FR5-treated groups were reduced (Figure 8A), with the most obvious reduction of tumor weight observed in the 120 mg/kg FR5-treated group (Figure 8B). The tumor specimens were examined using immunohistochemistry, and results showed the level of Bcl-2 and YAP were down-regulated (Figure 8C, 8J, 8K), but the expression of PTEN and PI3K was upregulated in the FR5-treated groups (Figure 8C, 8H, 8I). To further confirm the increase of apoptosis in Bel7402 cells by FR5, TUNEL staining assays were performed. As shown in Figure 8D, TUNEL positive cells were significantly increased after the treatment of 120 mg/kg FR5, compared with control (Figure 8F). As expected, FR5 inhibits the growth Bel7402 cell xenograft tumors in nude mice through PI3K/AKT pathway.

## DISCUSSION

*Marsdenia tenacissima*, a traditional herbal medicine, has various pharmacological effects and provides alternative treatment options for cancer patients [5–9]. Although MTE is a common herbal drug for adjuvant therapy after chemotherapy and radiotherapy, its side effects in cancer patients often occurs. To address this, the present study aimed to refine MTE to FR5 to improve anti-tumor efficacy and reduce toxicity. The research showed the strong anti-proliferation and pro-apoptosis effect of FR5 on HCC cells was achieved through inactivation of Hippo-YAP and PI3K/AKT and activation of PTEN signaling pathway. This study thus not only identifies the effect of FR5 on the PTEN/PI3K/AKT pathway, but also reveals a novel Hippo-YAP pathway for the effects of FR5 on HCC therapy.

Nowadays, herbal medicines or their derived compounds from them used in cancer patients are becoming increasingly popular. Plant-derived agents are an important source for cancer therapy, because they are more structurally diverse and “biologically friendly” molecular compared with synthetic compounds [28], e.g., paclitaxel, which is purified from the crude extraction of *pacific yew tree* [29]. MTE is an effective and complex drug, and it has more than forty C21 steroidal glycosides and various kinds of other compounds [10]. Previous studies had focused on the purification and structural analysis of monomers to reduce the side effects of MTE [10]. Ying et al. [30, 31] have obtained a variety of monomeric compounds from a fungal endophyte of Huperzia serrata. We cooperate with them to obtain the most active components in MTE and identify the five major components and examine them by HPLC (Figure 1C). Multiple functional monomers with similar structures can improve efficacy for the polymers [32]. The main components of FR5 are five representative C21 steroids, including Tenacissoside G, I, and Marsdenoside A, B and C [18, 19]. All of them have similar monomer structure and have synergistic effects on tumor treatment.

Previous studies had verified the anti-tumor effect of MTE on human acute T cell leukemia cells [9], and non-small cell lung cancer cell line [14]. In our study, the Bel7402 and HepG2 cells were used to evaluate the anti-cancer activity of FR5 in the *in vitro* and *in vivo* experiments. As we expected, the treatment of FR5 inhibited proliferation of Bel7402 and HepG2 cells in a dose and time dependent manner (Figure 2A, 2B). What makes us feel excited was the effect of synergy found between FR5 and PI3K inhibitor at the concentrations lower than IC50 (Figure 2C, 2D), which indicated FR5 at relative low concentrations had the potential in combination with other chemotherapy drugs. Meanwhile, we confirmed that FR5 significantly induced the apoptosis of HCC cells based on Annexin V-FITC/PI-stained flow cytometry (Figure 3) and TUNEL staining assays (Figure 8D). To shed the molecular mechanism of apoptosis induced by FR5, we investigated Bcl-2 and Bax by Q-PCR (Supplementary Figure 5), the downstream apoptosis regulators of PI3K/AKT pathway, serve as critical regulators of the mitochondrial-dependent apoptotic pathway [33]. Interestingly, we found that FR5 treatment decreased the expression of Bcl-2 and increased Bax level in Bel7402 cells (Figure 5D). Consistently, we also found that FR5 treatment decreased the expression of Bcl-2 based on immunohistochemical analysis of the Bel7402 xenograft tumors (Figure 8C), which suggested that the enhanced apoptosis of HCC cells by FR5 treatment may be due to the activation of mitochondrial apoptotic pathways. We also found that FR5 significantly inhibited migration of Bel7402 and HepG2 cells (Figure 4), which indicating that herbal medicine and its extracts can be used in conjunction with chemotherapy drugs in the inhibition of metastasis of HCC.

To elucidate the molecular mechanisms of FR5 on HCC cells, several growth factors and proteins were investigated in FR5-treated Bel7402 and HepG2 cells. PTEN modulates the downstream signaling pathway of PI3K/AKT, which plays a critical role in cell cycling, protein translation, and metastasis by AKT-mediated phosphorylation [21]. Overexpression of phosphorylated AKT was associated with disease-free survival, poor overall survival, and high tumor recurrence [22], which makes it an attractive target in cancer therapy. PI3K inhibitors impair the proliferation and survival of HCC cells [25]. It has been reported the inhibition of PI3K/AKT/mTOR pathway by MTE decreased the growth of gefitinib resistant non-small lung cancer cells [14]. The present study investigated the effect of FR5 on the PTEN/PI3K/AKT pathway. To our delight, these results indicated FR5 treatment increased the level of PTEN (Figure 5B) and decreased p-AKT(S473)/AKT and p-PI3K/PI3K (Figure 5C), which is consistent with the results of the immunohistochemical analysis of PTEN and PI3K (Figure 8C). Further, PI3 kinase inhibitor LY294002 enhanced the FR5-induced apoptosis and the inhibition of proliferation of Bel7402 and HepG2. All results indicated the PTEN/PI3K/AKT pathway mediates the effects of FR5 on HCC cells.

Recent studies have demonstrated crosstalk between the Hippo and PI3K-mTOR pathways in Drosophila, and identified PTEN as a critical mediator of YAP in mTOR regulation [17, 26, 27]. YAP, a direct downstream effector of the tumor suppressive Hippo pathway, has been reported to form a complex with sequence-specific DNA-binding proteins and regulate the expression of target genes that drive cell proliferation and inhibit apoptosis [20]. YAP mediates the effects of the Hippo pathway by regulating the expression of miR-29 family genes, which inhibit PTEN by targeting its 3′UTR [17]. Several studies have reported that YAP mediates cancer cell sensitivity to a number of therapies, including cisplatin, taxol, EGFR tyrosine kinase inhibitor, or small molecule inhibitor of surviving [34–36]. The effect of *Marsdenia tenacissima* on YAP in HCC had not been reported, which makes it a novel anti-metastatic target for the FR5 treatment for HCC. Based on the above evidence, the present study investigated whether FR5 induced apoptosis and inhibited proliferation of HCC cells is mediated through crosstalk between the Hippo and PI3K/AKT pathways via PTEN. For the first time, we found that FR5 increased the phosphorylation of YAP and decreased the expression of YAP (Figure 5A). In addition, YAP overexpression has been associated with the development of a variety of tumors, and is considered a tumorigenic gene, and overexpressed YAP proteins is detectable in more than half of HCC patients [36]. It was further examined if the anti-cancer effects of FR5 occur in YAP knockdown cells. It was found that YAP knockdown partly abolished FR5-induced regulation of PTEN and downregulation of p-AKT(S473) of Bel7402 and HepG2 cells treated by FR5 (Figure 7). Collectively, these results implied that FR5 induced anti-cancer effect through coordination between the Hippo and PI3K/AKT pathways, and this is executed at least in part through the actions of YAP and PTEN, which subsequently affects AKT, resulting in the eventual decrease in cell proliferation and increase in cell apoptosis on HCC cells.

In summary, the present study confirmed the anti-proliferative and pro-apoptosis effects of FR5 in HCC cells and propose a working model depicted in Figure 8E. FR5 not only affected PTEN/PI3K/AKT pathway but also Hippo pathway and then resulted in the suppression of HCC growth. Our results revealed a novel pathway of Hippo-YAP in associated with effects of FR5 on HCC and defined the mechanism. It is expected that FR5 may serve as an effective anti-tumor agent in the prevention and treatment of HCC.

## MATERIALS AND METHODS

### Plant material

The plant material used for this study was collected in Yunnan province of China and authenticated as *M. tenacissima* by Prof. Zha-Jun Zhan of Zhejiang University of Technology. A voucher specimen (No. ZJUT 1309MT) was deposited with the Zhejiang University of Technology.

### Preparation and chemical analysis of FR5

The dried stems of *M. tenacissima* (10 kg) were crushed and extracted with 95% ethanol (30 L × 4) at r. t. to give 987 g of extract (MTE), and each extraction time is 16-24h. The extracts were filtered and concentrated, followed by re-suspended in 2 L distilled water and partitioned with ethyl acetate (EtOAc) (0.5 L × 4) to furnish an EtOAc-soluble residue (570 g) after removing the solvent. The residue was subject to column chromatography on silica gel (9 × 80 cm) eluting with gradient petroleum ether and EtOAc (6:11:1) and petroleum ether and ethyl acetate (3:1) to afford eight fractions FR1-FR8. FR5 was analyzed by HPLC (Agilent 1200 Series, Agilent Technologies, USA) on a YMC Triart C18 column (250 × 4.6 mm, 5 μm, YMC CO., LTD., Japan) eluted with 80 % (v/v) methanol in water at a flow rate of 1.0 mL/min and detected at 210 nm. Compounds 1-5 were isolated from FR5 on preparative HPLC systems equipped with a Waters 600 pump, a Waters 2487 UV detector, a YMC-Pack R & D ODS-A C18 column (250 × 20 mm, 5 μm, YMC CO., LTD., Japan) and an N 2000 chromatography workstation. Samples of FR5 were eluted with 78 % (v/v) methanol in water at a flow rate of 4.0 mL/min and detected at 210 nm. The structure the five compounds were elucidated on the basis of nuclear magnetic resonance (NMR) analyses and comparison with the literatures.

### Cell culture

Bel7402 cell line was provided by Key Laboratory of Gastroenterology of Zhejiang Province, Zhejiang Provincial People's Hospital, Hangzhou, Zhejiang, China. HepG2 cell line was purchased from the Type Culture Collection of the Chinese Academy of Sciences, Shanghai, China. Bel7402 Cells and HepG2 cells were respectively grown in Roswell Park Memorial Institute-1640 (RPMI-1640, Gibco, Grand Island, NY, USA) and Roswell Park Memorial Institute-DMEM (DMEM/HIGH GLUGOSE(1X), Gibco, Grand Island, NY, USA) supplemented with 10% fetal bovine serum (FBS) and 100 U/mL streptomycin/penicillin at 37°C in atmosphere of 5% CO2. When reached to 80-90% confluences, Bel7402 cells and HepG2 cells were collected for subsequent experiments.

### Cell viability assay

The viability of cells was evaluated by a 3-(4, 5-dimethylthiazol-2-yl)-5(3-carboxymethoxyphenyl) -2-(4-sulfopheny)-2H- tetrazolium, inner salt (MTS) assay. Cells were seeded in 96-well plates at a density of 5×10^3^ per well, and incubated for 24 h. Hepatogenic non-cancerous cell (L02 cell) and renal non-cancerous cell (HEK293 cell) were used as normal cell control. Cells were exposed to various concentrations (0, 20, 40, 80, 160, and 320 ug/ml) of FR5 for 24 h and 48h with three replicates for each testing point including control group (containing 0.1% DMSO) and blank wells. Thereafter, cells were incubated with MTS (1:5) (Promega, St. Louis, MO, USA) for 2 h at 37°C. Optical density (OD) values were measured at 490 nm in a microplate reader (BIOTEK, Vermont, USA). The results are expressed as cell viability rates.

### Cell apoptosis assay

Both exponentially growing HepG2 or Bel7402 cells were seeded into 12-well culture plates and incubated for 24 h, followed by a treatment of different concentrations (0,80 and 160 μg/mL) of Fr5. After 24h and 48h of treatment, all cells were collected and re-suspended in 500 μL binding buffer and sequentially mixed with 5 μl Annexin V-FITC and 5 μl PI. After 15min of incubation at room temperature in the dark, a flow cytometric analysis was performed with a flow cytometry (Becton-Dickinso, Franklin Lakes, USA) and the quantitation of apoptotic cells was calculated by CellQuest software.

### Cell migration assay

The migration assay was performed with Transwell plates (3422; Corning Incorporated, Corning, NY, USA) containing a membrane with 8 μm pores. Cells (5×10^5^ cells for migration assays) were re-suspended in serum-free medium and seeded into the upper chamber. Different concentrations (0, 40, 80 ug/ml) of FR5 were loaded in the upper chambers, and culture medium containing 20% fetal bovine serum was added to the lower chamber as the chemoattractant. The cells were incubated in a humidified incubator at 37°C for 24 h. Non-invading cells in the upper chambers were removed with cotton swabs. The cells attached to the lower surface were fixed and stained. The number of cells attached to the lower surface were counted in five random fields under a microscope (×200).

### Cell transfection

Bel7402 and HepG2 were cultured in RPMI-1640 and DMEM containing 10% FBS and 50 μg/mL penicillin/streptomycin (P/S) respectively. YAP knockdown cells were generated by lentiviral infection as previously described [37]. Plasmids were propagated in and purified from Stbl2 competent cells (Invitrogen). For virus production, HEK293T cells were transfected with lentiviral constructs and packaging plasmids (psPAX2 and pMD2.G). Forty-eight hours after transfection, the lentiviral supernatant was supplemented with 0.5 g/mL polybrene, filtered through a 0.45-M filter, and used to infect target cells. Thirty-six hours after infection, Cells were selected with 5 μg/mL puromycin in culture medium. Cells were treated with FR5 at the concentration of 160 μg/ml for at least 48 h after transfection and Western Blot analysis was performed. YAP shRNAs were described previously [38].

### Western blotting analysis

Proteins were extracted by lysing Bel7402 and HepG2 cells with RIPA lysate (Beyotime) containing 1% phenylmethanesulfonyl fluoride (PMSF, Beyotime). The concentration of proteins was quantitated using a bicinchoninic acid (BCA) protein assay kit (Beyotime). Equal amounts of proteins were loaded and separated by SDS-polyacrylamide gel electrophoresis (PAGE) followed by electrotransferred onto polyvinylidene fluoride (PVDF) membranes (Millipore, Bedford, MA, USA). The blots were blocked with 5% non-fat milk for 1 h and probed with specific primary antibodies against YAP (Santa Cruz Biotechnology, Delaware, USA), p-YAP, PTEN, PI3K, p-PI3K, AKT, p-AKT(Ser473), p-AKT(T308) (all Cell Signaling Technology, Danvers, MA, USA) and Bcl-2, Bax (all HuaAn Biotechnology, Hangzhou, China) at 4°C overnight and incubated subsequently at 37°C with their corresponding secondary antibodies (Beyotime, Haimen, China) for 45 min. Unbound antibodies in each step were washed by TBST three times. Target bands were visualized by enhanced chemiluminescence (ECL) solution (Qihai Biotec, Shanghai, China) and measured by Gel-Pro-Analyzer software (Bethesda, MD, USA). GAPDH was employed as an internal control.

### *In vivo* xenograft model assay

Fifteen male BALB/C nude mice aged 4 weeks (Shanghai SLAC Laboratory Animal Center of Chinese Academy of Sciences, Shanghai, China) were used to establish HCC xenograft model. Bel7402 cells, at 2 × 106 suspended in 100 μl of PBS, were implanted by subcutaneous injection into the right flanks of mice. After a week, mice were randomly divided into three groups (n = 5 per group). Two groups were injected with FR5 at doses of 60 and 120 mg/kg, respectively, which was dissolved in 0.2 mL of 0.9% saline solution by intravenously daily for a period of 10 days. Mice in the control group were injected with normal saline. The tumors were regularly measured with calipers and the tumor volume was estimated by the equation “0.5 × a × b2”, in which a and b represents the maximal and minimal diameters, respectively. The tumor inhibition ratio (%) was calculated as follows: Tumor inhibition ratio (%) = [(C-T)/C] × 100%, where C is the tumor weight average of the blank control (normal saline), and T is that of the treated group. After treatment, all mice were sacrificed and the tumors were harvested and weighed. The paraffin-embedded sections (5 μm thick) were prepared for examining the expressions of Bcl-2, PI3K (Servicebio, Wuhan, China), PTEN, and YAP by immunohistochemistry with the streptavidin-peroxidase (S-P) kit (Fuzhou Maixin Biotechnology Development Co., Fuzhou, China). The percentages of positive cells were counted within 5 high-power fields.

### *In situ* apoptosis detection by TUNEL staining assay

A fluorescent *in situ* terminal deoxynucleotidyltrans-ferase-mediated nick end labeling (TUNEL) assay was performed using an *in situ* apoptotic cell detection kit (Boehringer Mannheim, Indianapolis, USA) following the manufacturers protocol. Paraffin-embedded, 5 μm thick tumor sections were used to identify apoptotic cells by staining using a TUNEL assay. It is based on the enzymatic addition of digoxigenin nucleotide to the nicked DNA by terminal deoxynucleotidyl transferase. The extent of apoptosis was evaluated by counting the TUNEL-positive cells. The apoptotic index was determined as a number of TUNEL-positive cells/total number of cells in 5 randomly selected high power fields (magnification × 200).

### Statistical analysis

Statistical analysis was performed with GraphPad Prism 6.0 software. All data were presented in bar graph formats and given as mean ± standard deviation (SD). Differences between groups were analyzed with one-way analysis of variance (ANOVA); p<0.05 was considered to be statistically significant.

## SUPPLEMENTARY MATERIALS FIGURES AND TABLE


