# Supplementary material for: C21 steroid-enriched fraction refined from Marsdenia tenacissima inhibits hepatocellular carcinoma through the coordination of Hippo-Yap and PTEN-PI3K/AKT signaling pathways
Source: Oncotarget. 2017 Nov 30;8(66):110576–91. doi: 10.18632/oncotarget.22833 (PMC5746405; doi:10.18632/oncotarget.22833)
Supplement: Supplementary file 1 [file oncotarget-08-110576-s001.pdf]

C21 steroid-enriched fraction refined from *Marsdenia tenacissima* inhibits hepatocellular carcinoma through the coordination of Hippo-Yap and PTEN-PI3K/AKT signaling pathways

SUPPLEMENTARY MATERIALS

Cell viability assay

The viability of Bel7402 cells was evaluated by a MTS assay. Cells were seeded in 96-well plates at a density of 5×10^3 per well, and incubated for 24 h. Cells were exposed to various concentrations (0, 80, 160, 320 and 640μg/ml) of Xiao-ai-ping injection for 24 h with three replicates for each testing point including blank wells. Thereafter, cells were incubated with MTS (1:5) (Promega, St. Louis, MO, USA) for 2 h at 37 °C. Optical density (OD) values were measured at 490 nm in a microplate reader (BIOTEK, Vermont, USA). The results are expressed as cell viability rates.

Real-time quantitative reverse transcription-PCR (RT-qPCR)

Cells in each sample were grown in coverslips with appropriate treatment before total RNA was extracted by an RNA extraction kit (Invitrogen, Carlsbad, CA, USA). 50 ng RNA was reverse-transcribed into cDNA. RT-qPCR reactions were carried out by a real-time PCR device (MiniOpticon, Bio Rad, China) using SYBR Green Super Mix (Solarbio, Beijing, China) with the primers sequences in Supplementary Table 1. Relative mRNA expression was calculated by 2 -ΔΔCT method and β-actin was used as an internal control.

Western blotting analysis

Proteins were extracted by lysing Bel7402 cells with RIPA lysate (Beyotime) containing 1% phenylmethanesulfonyl fluoride (PMSF, Beyotime). Equal amounts of proteins were loaded and separated by SDS-polyacrylamide gel electrophoresis (PAGE) followed by electrotransferred onto polyvinylidene fluoride (PVDF) membranes (Millipore, Bedford, MA, USA). The blots were blocked with 5% non-fat milk for 1 h and probed with specific primary antibodies against YAP (Santa Cruz Biotechnology, Delaware, USA) at 4 °C overnight and incubated subsequently at 37 °C with their corresponding secondary antibodies (Beyotime, Haimen, China) for 45 min. Unbound antibodies in each step were washed by TBST three times. Target bands were visualized by enhanced chemiluminescence (ECL) solution (Qihai Biotec, Shanghai, China) and measured by Gel-Pro-Analyzer software (Bethesda, MD, USA). GAPDH was employed as an internal control.

Real time celluar analysis profiling

Bel7402 and HepG2 cells were seeded at 2500 cells per well onto an E-Plate (Roche Diagnostics GmbH) and monitored using the xCELLigence system (Roche Diagnostics GmbH, Switzerland). After 20 h of Real Time Celluar Analysis (RTCA) profiling, the cells were treated with FR5 prediluted in growth media to a final concentration of 40, 80 and 160 μg/ml, and responses were measured at 15-min intervals for 72 hours.

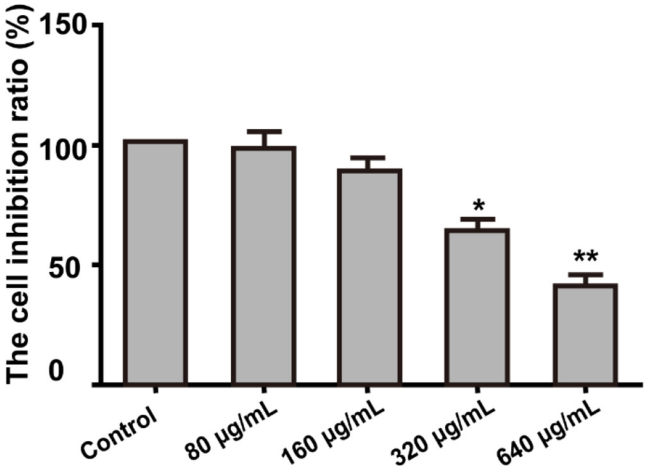

Supplementary Figure 1: Effect of Xiao-ai-ping injection on the proliferation of Bel7402 cells. Bel-7402 cells were treated with Xiao-ai-ping injection at concentrations of 0, 80, 160, 320 and 640μg/ml for 24h; the inhibition rates were examined using MTS assays. The data are expressed in terms of percent of control cells as the means ± SD. The experiments were repeated at least three times. $^{*}p<0.05$; $^{**}p<0.01$ vs. control group.

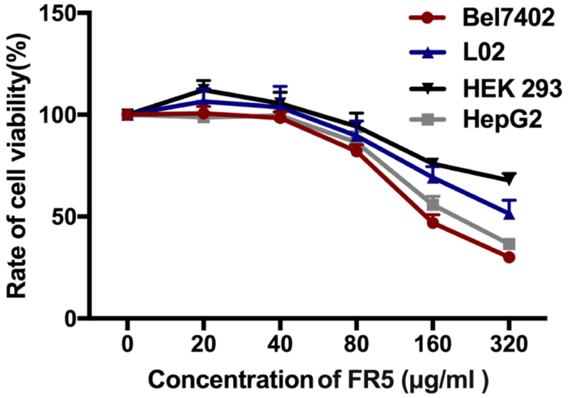

Supplementary Figure 2: Effect of FR5 on the proliferation of non-cancerous cells. Hepatogenic non-cancerous cell (L02 cell) and renal non-cancerous cell (HEK293 cell) were used as normal cell control. Bel7402, HepG2, HEK293 and L02 cells were treated with FR5 at concentrations of 0, 20, 40, 80, 160 and 320 μg/ml for 24 h; the cell viability rates were examined.

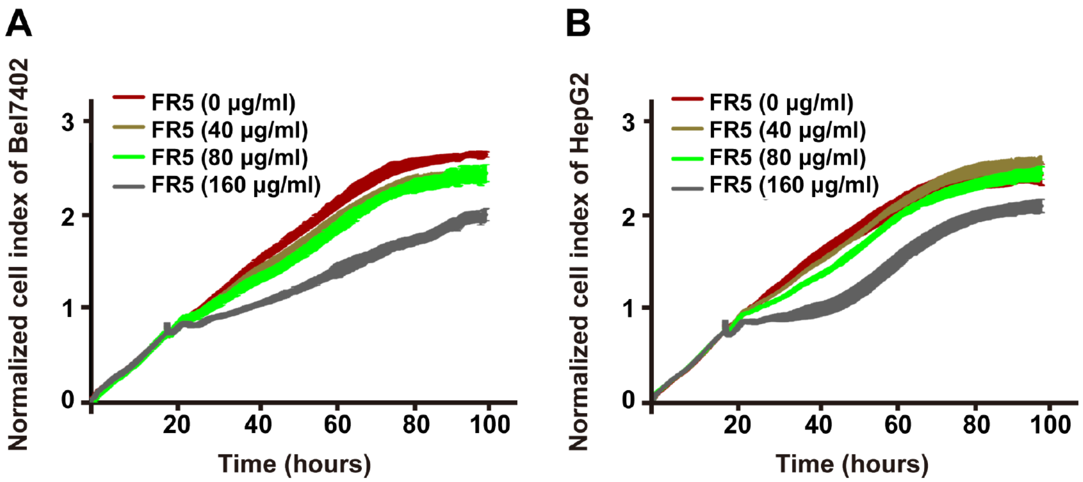

Supplementary Figure 3: RTCA profiling of FR5 on the proliferation of Bel7402 and HepG2 cells. Bel7402 and HepG2 cells were seeded at 2500 cells per well onto an E-Plate and were monitored overnight and then treated with FR5 at concentrations of 40, 80 and 160 μg/ml at approximately 20 h, and responses were measured at 15-min intervals for 72 hours.

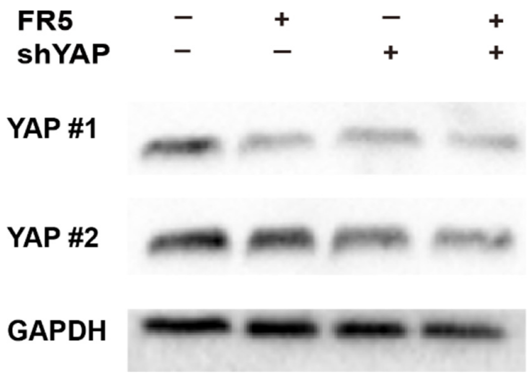

Supplementary Figure 4: Effect of YAP1/2 shRNAs on the depletion of YAP protein expression. Bel7402 cells were infected with the indicated shRNA lentiviruses, and the levels of YAP protein were determined in control and FR5-exposed Bel7402 cells transfected with YAP shRNA or control shRNA by western blot. GAPDH was used as internal control.

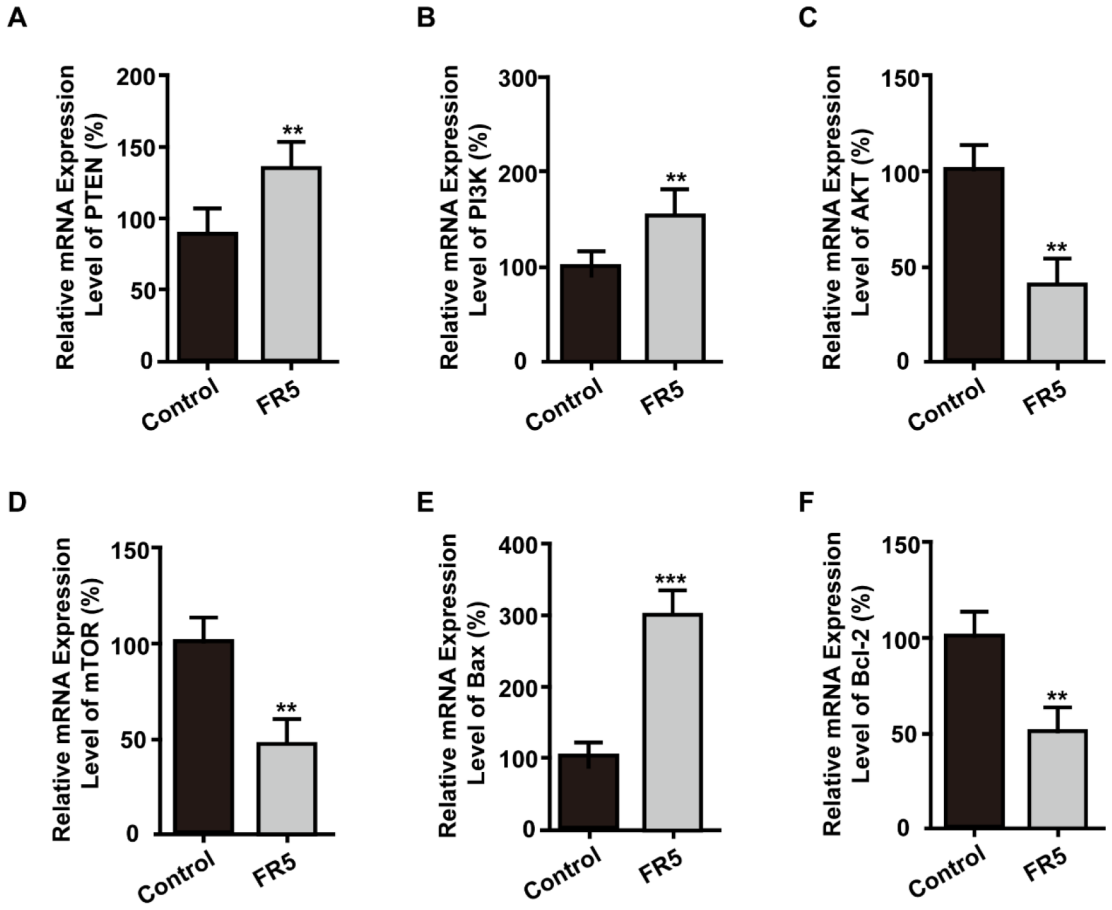

Supplementary Figure 5: FR5 inhibits PI3K/AKT/mTOR pathway and enhances PTEN pathway in Bel7402 cells. Bel7402 cells were incubated with FR5 at the concentrations of 0 (Ctrl) and 160µg/ml for 24 h, and RT-qPCR assays were carried out to investigate the mRNA levels of indicated genes. **(A)** The mRNA expression levels of PTEN. **(B)** The mRNA expression levels of PI3K. **(C)** The mRNA expression levels of AKT. **(D)** The mRNA expression levels of mTOR. **(E)** The mRNA expression levels of Bax. **(F)** The mRNA expression levels of Bcl-2. corresponding densitometric analysis is presented on the right as mean ± SD. Experiments were performed for three times, β-actin was used as internal control, and data are presented as mean ± SD. $^{**}p<0.01$; $^{***}p<0.001$ vs control group.

Supplementary Table 1: Sequences of primers for real-time quantitative reverse transcription-PCR

Primer name	Sequence (5'¬3')
β-actin F	TGAACGGGAAGCTCACTGG
β-actin R	TCCACCACCCTGTTGCTGGA
Bcl-2 F	GGATCCAGGATAACGGAGGC
Bcl-2 R	CCAGATAGGCACCCAGGGT
Bax F	CCCGAGAGGTCTTTTTCCGAG
Bax R	CCAGCCCATGATGGTTCTGAT
PI3K-CA-F	AGTAGGCAACCGTGAAGAAAAG
PI3K-CA-R	GAGGTGAATTGAGGTCCCTAAGA
PTEN F	ACCAGGACCAGAGGAAACCT
PTEN R	GCTAGCCTCTGGATTTGACG
AKT F	TAGGTGTGGATGGTTGCAGA
AKT R	CTCCCACAGACCCAGAACAT
mTOR F	TCCGGCTGCTGTAGCTTATTA
mTOR R	CGAGCATATGCCAAAGCACT
